# Identifying risk factors in explaining women’s anaemia in limited resource areas: evidence from West Bengal of India and Bangladesh

**DOI:** 10.1186/s12889-022-13806-5

**Published:** 2022-07-28

**Authors:** Arup Jana, Aparajita Chattopadhyay, Unnati Rani Saha

**Affiliations:** 1 Research Scholar, Department of Population & Development, International Institute for Population Sciences, Deonar Mumbai, India; 2 Department of Population & Development, International Institute for Population Sciences, Mumbai, India; 3 Department of Public Health, Erasmus MC, University Medical Center Rotterdam, Rotterdam, the Netherlands

**Keywords:** Anaemia, Reproductive age, Female sterilization, Religion, WASH, India West Bengal, Bangladesh

## Abstract

**Background:**

Anaemia among women is a public health problem with associated adverse outcomes for mother and child. This study investigates the determinants of women’s anaemia in two Bengals; West Bengal (a province of India) and Bangladesh. These two spaces are inhabitated by Bengali speaking population since historic past. The study argues that open defecation, contraceptive method use and food consumption patterns are playing crucial role in explaining anaemia.

**Methods:**

Using non-pregnant women belonging to different religious groups, we analyzed a total of 21,032 women aged 15–49 from the nationally representative cross-sectional surveys, i.e., Bangladesh Demographic Health Survey (BDHS-VI, 2011) and National Family Health Survey (NFHS round 4, 2015–16). We performed spatial, bivariate and logistic regression analyses to unfold the important risk factors of anaemia in two Bengals.

**Results:**

The prevalence of anaemia was 64% in West Bengal and 41% in Bangladesh. The significant risk factors explaining anaemia were use of sterilization, vegetarian diet and open defecation. Further, women who used groundwater (tube well or well) for drinking suffered more from anaemia. Also, younger women, poor, less educated and having more children were highly likely to be anaemic. The study also indicates that those who frequently consumed non-vegetarian items and fruits in West Bengal and experienced household food security in Bangladesh were less prone to be anaemic. Hindus of West Bengal, followed by Muslims of that state and then Hindus of Bangladesh were at the higher risk of anaemia compared to Muslims of Bangladesh, indicating the stronger role of space over religion in addressing anaemia. Unlike West Bengal, Bangladesh observed distinct regional differences in women's anaemia.

**Conclusions:**

Propagating the choices of contraception mainly Pill/ injection/IUDs and making the availability of iron rich food along with a favourable community environment in terms of safe drinking water and improved sanitation besides better education and economic condition can help to tackle anaemia in limited-resource areas.

**Supplementary Information:**

The online version contains supplementary material available at 10.1186/s12889-022-13806-5.

## Introduction

Anaemia is a global public health problem that affects mainly young children and adult women. About 1.71 billion people, or 23% of the global population [1], and approximately one-third of the women in childbearing age [2] suffer from anaemia. Based on the level of anaemia as a public health concern, the World Health Organization (WHO) ranks India third in severity (more than 50% of women suffer from anaemia). At the same time, neighbouring countries such as Bangladesh, Sri Lanka, and Nepal perform better than India in this ranking. The prevalence of anaemia in Bangladesh reduced from 50.3% in 1990 to 39.7% in 2015 [3], while during this period, the decline in anaemia was insignificant in the state of West Bengal of India [4]. India allocates 3.6 per cent of its Gross Domestic Product (GDP) to public health [5], while the country suffers a 3% GDP loss due to the burden of Iron Deficiency Anaemia (IDA) in children [6].

Anaemia in the reproductive age of women is defined as the haemoglobin level of less than 11 g per decilitre [7]. The deficiency of micronutrients like iron, zinc, vitamin B12, vitamin A, and folic acid is linked to inadequate nutrient consumption and is the primary predictor of anaemia [8–13]. In addition, large-scale studies have found that low socioeconomic status and lack of education are major determinants of anaemia in women [14–16]. Uneducated women have poor knowledge of the quality and nutritional content of the foods they consume [17]. Thus, women with lower socioeconomic status are prone to be undernourished and anaemic condition due to inadequate food consumption [18, 19]. Moreover, unhygienic practices and a lack of improved drinking water also directly affect the bioavailability of the food consumed [20]. High fertility, physical work, parasite infections, and menstrual disorders are similarly responsible for anaemia in women [21, 22]. Further, female sterilization and anaemia are strongly associated, though studies are limited in India and Bangladesh [23–26].

Anaemia is a leading factor in high maternal morbidity and mortality [27]. However, it also impacts development [6, 28], being the causal factor of adverse birth outcomes, low birth weight and preterm birth [29], and cognitive impairment, depression [30–32] and work productivity loss [33]. Also, anaemia leads to 11% of Years Lived with Disability (YLDs) [34]. More than 1,15,000 maternal deaths and 5,91,000 perinatal deaths occur each year due to anaemia [35].

Anaemia Mukt Bharat (anaemia free India) was launched recently by the Government of India to reduce the prevalence of anaemia by 3% points yearly among children, adolescents, and women in the reproductive age (15–49 years) from 2018 to 2022 [36]. However, over the past decade, a drop of mere 0.7 percentage points was experienced by women (from 62.5% in 2006 to 61.8% in 2016) in West Bengal, indicating the failure of these targets [4]. At the same time, a yearly reduction in the prevalence of anaemia by 1.1 percentage points was observed in Bangladesh [3].

Therefore, the rationale for this research is to understand the factors explaining anaemia in two Bengals: West Bengal and Bangladesh. Also, interest lies in identifying the spatial clustering of anaemia in two neighbouring spaces. These two geographical areas are identical in ethnic, environmental and linguistic parameters; while the religious composition differs significantly. Bangladesh had a Muslim majority (89%), while West Bengal had a Hindu majority (75%). To our knowledge, there is no comparative study on predictors of anaemia among women of childbearing age in West Bengal and Bangladesh with a special emphasis on the religious composition. It is hypothesized that open defecation (West Bengal 39%, Bangladesh: 1%), women’s sterilization (West Bengal: 36%, Bangladesh: 5%), and food consumption patterns that are significantly different in these two places play a crucial role in explaining anaemia. Thus, it would be helpful for the policymakers to relook into the strategies to achieve the second goal of SDGs, that is, fighting against malnutrition, especially in limited-resource countries.

## Methods

### Data sources

The present study used data from the fourth round of the National Family Health Survey (NFHS-4), 2015–16 (India), and from the Bangladesh Demographic Health Survey, 2011 (Bangladesh), for anaemia and associated risk factors. NFHS-4 was conducted from January 2015 to December 2016 across 36 states and union territories, including all the districts in West Bengal of India and provided information on various important Maternal and Child Health (MCH) indicators. In the NFHS dataset, blood samples were collected from 17,093 out of 17,668 women. The haemoglobin concentration was measured using a battery-operated portable HemoCue Hb 201 + analyzer. The Bangladesh Demographic Survey (BDHS-VI, 2011) was conducted from June to November 2011 and is the latest data set for Bangladesh, where information on anaemia is available. Blood samples were collected from children, men, and women – both pregnant and non-pregnant. One-third of households were selected for anaemia testing in Bangladesh. Since the recent BDHS-VII, 2017-18 does not provide information about anaemia, the most recent survey (BDHS-VI, 2011) was used in the study, which provides data on anaemia.

A two-stage stratified sampling design was adopted in both data sets mentioned above. Census enumeration areas or 'clusters' were selected as the primary sampling unit at the first stage of the sampling frame. In the second stage, households were randomly selected from the primary sampling units or 'clusters' of the sampling frame (see for Bangladesh, NIPORT et al., 2011, and for India, IIPS & ICF, 2016 [4, 37]).

### Sample selection

The study restricted the sample to two religious groups – Hindu and Muslim—focusing on non-pregnant women. It excluded pregnant women to avoid selection bias as food consumption and BMI indicators vary between pregnant and non-pregnant groups [38]. A total of 17,092 women participated in the survey in the Indian state of West Bengal. Among them, 660 pregnant women and 604 samples belonging to other religions were excluded. Hence, 15,756 samples were taken for the final analysis after excluding the missing data.

In BDHS, though the total sample was 17,842 women aged 15–49, blood samples were collected for 5,983 women, of which 382 were identified as pregnant. We further excluded 32 samples belonging to other religious groups. The final sample was 5,276 women.

### Outcome variable

The DHS provides the altitude haemoglobin level. The WHO cut-off was followed to estimate anaemia among women. Anaemic women were defined as those who had less than 12 g/DL haemoglobin levels. For the analysis, anaemia was taken as a dichotomous variable, where 0 signifies not anaemic, and 1 denotes anaemic.

### Predictor variables

The predictor variables were selected based on the previous studies and available variables in the dataset. Independent variables considered as covariates in this study were indicators of different levels of socioeconomic, demographic variables, health status, and food consumption/security. As BDHS does not provide the food consumption variable, food security was considered as a proxy of household food availability. Variables considered for women were age, nutritional status, method-mix in contraceptive use, religious affiliation (Hindu vs Muslim), and education. Age was categorized as 'below 20', '20–24', '25–29' and '30 & above'. Body Mass Index (BMI) was categorised into 'thin' (< 18.5 kg/m^2^), 'normal' (18.5–24.99 kg/m^2^), and 'overweight or Obese' (≥ 25 kg/m^2^). Educational qualification was considered in the model as a proxy for the social status of the household. As women's reproductive behaviour can influence their health outcomes, the current contraceptive method uses and the number of children born to a woman were taken into account. The contraceptive methods was categorized into 'no use', 'female sterilization', 'pill/injection/intrauterine device (IUD)', and 'other'.

The wealth quintile represents a household's economic condition; it was calculated on standardized wealth scores given in the DHS datasets. The residential/community environment was captured through the region, urban–rural residence and ownership of agricultural land and open defecation. The sources of drinking water were classified as 'groundwater' (tube well or well), 'distributed water' (tank, tap, and piped water), and 'other' (bottled, spring, and surface water).

The World Food Summit in 1996 defined food security as 'when all people, at all times, have physical and economic access to sufficient safe and nutritious food to meet their dietary needs and food preferences for a healthy and active life' [39]. The food security index was calculated following the methodology of Chowdhury et al., 2018 [40]. Food consumption indicators of NFHS were categorized as 'never', 'daily, 'weekly/occasionally', etc., based on the type of food and its consumption frequency. This study categorized the administrative divisions of West Bengal and Bangladesh as 'regions'.

### Statistical analysis

The outcome measure, anaemia, was categorized into '1' if a woman had anaemia and ‘0’ otherwise. A Chi-square test was used to mark the significance levels of the unadjusted covariate's effect on anaemia.

Ordinary kriging, a linear geo-statistical interpolation technique, was used to prepare the spatial prevalence map of anaemia for creating cluster-level maps. For making these maps, the GPS coordinates were obtained from the DHS survey, with the rural clusters displaced 5 kms and urban clusters displaced 2 kms to maintain confidentiality. Univariate Moran's I statistics technique was applied to identify the hot spots and the cold spots of anaemia among women in Bangladesh and West Bengal. Bivariate Moran's I is a spatial technique that measures the autocorrelation to assess an independent variable's influence on a dependent variable. Bivariate Moran's I was employed using the cluster points of DHS data to understand the spatial association between anaemia and groundwater.

Furthermore, a multiple logistic regression analysis was applied to capture the differences in the covariate's effect on the outcome measure on two spatial units separately and then on the pooled data. In the pooled data, analysis was made with interaction terms involving the dummy of two spatial units and the religion categories. The analyses were adjusted for sampling weight. We checked multi-collinearity using the Variance Inflation Factor (VIF) for each independent variable included in the logistic regression. We did not find evidence of multi-collinearity as the VIF value was less than 2. Statistical analysis was performed using STATA version 14.1, while the spatial analysis was done using ArcMap version 10.3 and GeoDa version 1.18.

## Results

### Sample characteristics

The sample (Table 1) of West Bengal encompasses more adolescents (17%) compared to the sample of Bangladesh (10%). The use of contraceptives differed between the two places, with sterilization of women being five times more prevalent in West Bengal (26% in West Bengal vs 5% in Bangladesh), while more than a double proportion of women were using hormonal methods in Bangladesh (40% in Bangladesh vs 17% in West Bengal). Twenty-four per cent of women in West Bengal had no children compared to 8% in Bangladesh. On the other hand, nearly a half and one-fourth of the women respectively in Bangladesh and West Bengal had more than two children. Bangladesh had more illiterate women (57%) than West Bengal (44%). However, it is interesting to note that Bangladesh had an improved households' (HHs) wealth score and the proportion of rich households was higher in Bangladesh (45% vs 23%), while that of poor households was more in West Bengal (55 vs 36%). It was further observed that the people of West Bengal practiced open defecation about 10 times more than those of Bangladesh. Thirty-five per cent of all households in Bangladesh suffered from food insecurity. Ownership of agricultural land was higher in Bangladesh (47%) compared to West Bengal (34%). Sixty-five per cent of women in Bangladesh and 75% of women in West Bengal lived in rural areas. About half of the women consumed meat or chicken daily or weekly, while 56% of women never or occasionally consumed any fruit in West Bengal.

### Prevalence of anaemia: West Bengal vs Bangladesh

**Fig. 1:**
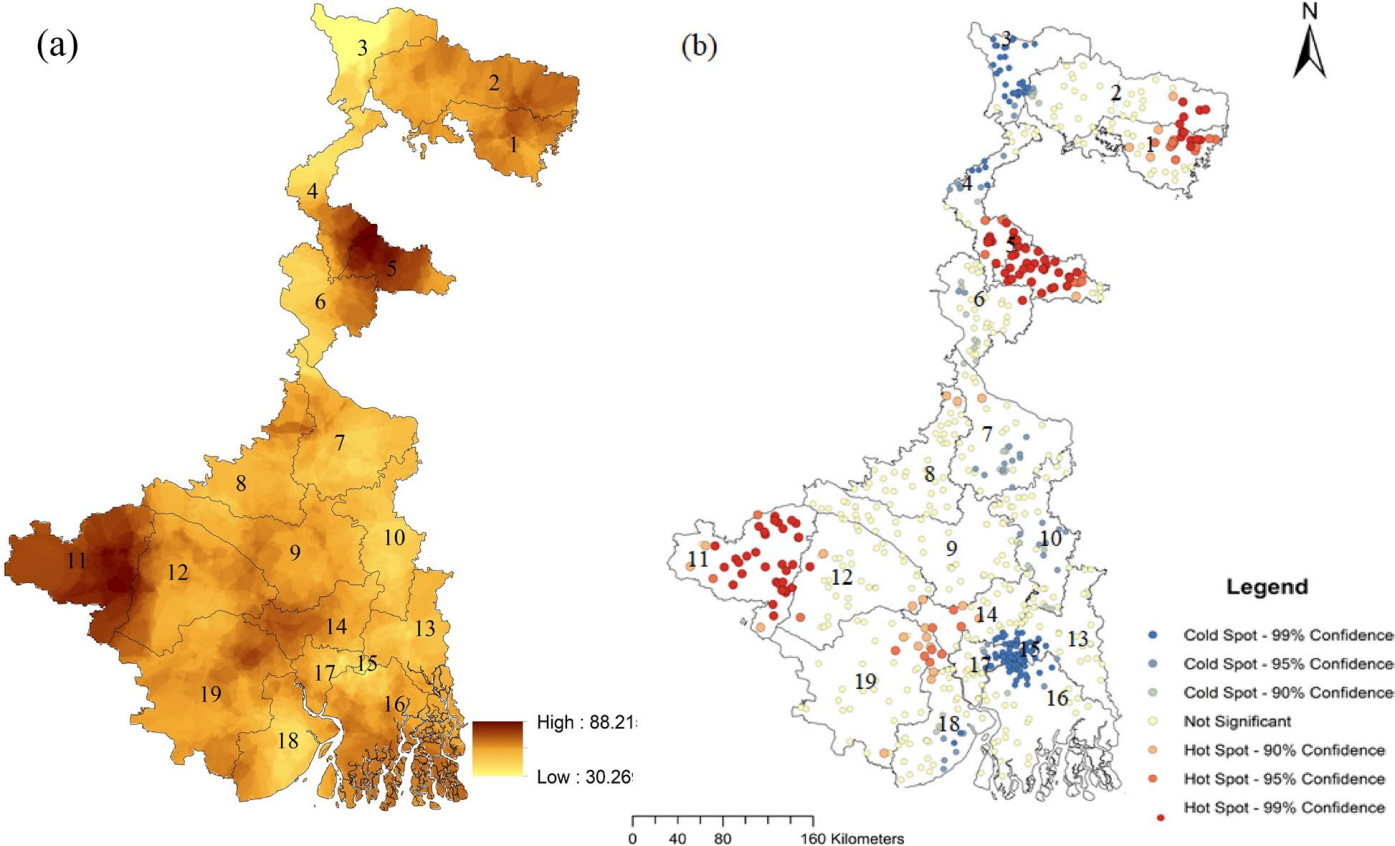
Spatial distribution (**a**) and hotspots (**b**) of the prevalence of anaemia among women in West Bengal. Note: 1. Koch Bihar 2. Jalpaiguri 3. Darjiling 4. Uttar Dinajpur 5. Dakshin Dinajpur 6.Maldah 7. Murshidabad 8. Birbhum 9. Barddhaman 10.Nadia 11. Puruliya 12. Bankura 13. North 24 Parganas 14. Hugl 15. Kolkata 16. North 24 Parganas 17. Haora 18. Purba Medinipur 19. Pashchim Medinipur

**Fig. 2:**
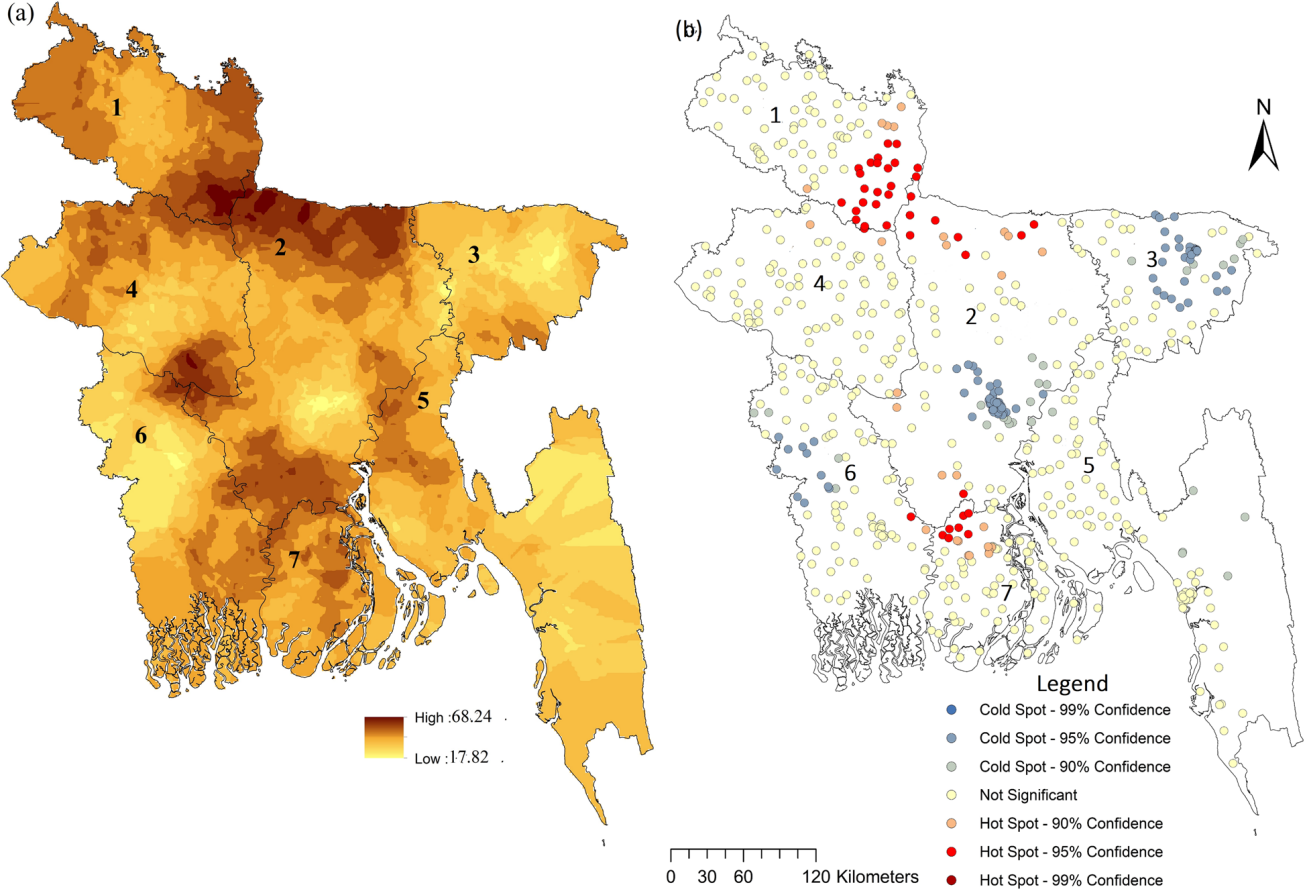
Spatial distribution (**a**) and hotspots (**b**) of the prevalence of anaemia among women in Bangladesh. Note: 1.Rangpur, 2. Dhaka, 3.Sylhet 4.rajshahi,5.chittagong, 6.Khulna 7.Barisal

The prevalence of anaemia was 64% in West Bengal and 41% in Bangladesh (Table 1). The hotspot analysis shows that the anaemia among women was highly concentrated in the western districts (Puruliya), followed by Dakshin Dinajpur and the north-eastern part of West Bengal (Fig. 1). In Bangladesh, the southern Rangpur, northern Dhaka and some parts of Barisal had a high concentration of anaemia (Fig. 2**)**. The severity of anaemia was distinctly more in different parts of West Bengal than in Bangladesh.

**Table 1:** Distribution of sample of West Bengal and Bangladesh: NFHS 2015-16IV and BDHS 2011

Variables	West Bengal		Bangladesh	
	Frequency (15,756)	Percentage	Frequency (5,276)	Percentage
**Anaemia**				
Yes	10,033	63.66	2,179	41.30
No	5,723	36.34	3,097	58.70
**Demographic and health factors**				
**Age**				
Below 20	2,616	16.60	534	10.12
20–24	2,652	16.83	939	17.80
25–29	2,452	15.56	958	18.16
30&above	8,041	51.02	2,845	53.92
**Body mass index**				
Thin	3,663	23.24	1,316	24.94
Normal	9,291	58.95	3,008	57.01
Overweight or Obese	2,807	17.81	952	18.04
**Current contraceptive method**				
Not using	6,623	42.02	2,026	38.40
Female sterilisation	4,097	25.99	255	4.83
Pill/injection\IUD	2,674	16.97	2,054	38.93
Others^a^	2,502	15.02	941	17.84
**Children ever born**				
No child	3,797	24.09	441	8.36
1–2	7,889	50.05	2,397	45.43
2 +	4,075	25.85	2,438	46.21
**Socioeconomic factors**				
**Education**				
Illiterate/primary	6,909	43.84	3,009	57.03
Secondary	7,805	49.52	1,872	35.48
Higher	1,047	6.64	395	7.49
**Religion**				
Hindu	11,790	74.80	592	11.22
Muslim	3,971	25.20	4,684	88.78
**Wealth**				
Poor	8,660	54.95	1,893	35.88
Middle	3,463	21.97	1,014	19.22
Rich	3,638	23.08	2,369	44.90
**Source of drinking water**				
Distributed water	10,702	29.48	581	11.01
Groundwater	4,645	67.91	4,225	80.08
Others	409	2.60	470	8.91
**Practicing open defecation**				
Yes	7,9914,623	70.67	177	3.35
No	11,138	29.33	5,099	96.65
**Has own land for agriculture**				
Yes	5,400	34.26	2,504	47.46
No	10,361	65.74	2,772	52.54
**Place of residence**				
Urban	4,297	27.26	1,847	35.01
Rural	11,464	72.74	3,429	64.99
**Food related factors**				
**Food security**				
Secure	NA	NA	3,396	64.71
Insecure	NA	NA	1,852	35.29
**Consume pulses**				
Daily	6,804	43.17	NA	NA
Never/weekly/occasionally	8,957	56.83	NA	NA
**Consume fruits**				
Daily	1,429	9.07	NA	NA
Never/occasionally	8,773	55.66	NA	NA
Weekly	5,559	35.27	NA	NA
**Consume chicken or meat**				
Daily/ weekly	8,122	51.53	NA	NA
Never	519	3.29	NA	NA
Occasionally	7,120	45.17	NA	NA
**Region**				
A	2,339	14.84	584	11.07
B	2,469	15.67	818	15.50
C	3,905	24.78	924	17.51
D	3,522	22.35	820	15.54
E	3,526	22.37	790	14.97
F			741	14.04
G			599	11.35

Administrative regions of west Bengal in the table A; Jalpaiguri, B;Burdwan, C;Presidency, D;Maldah, E;Medinipur and in Bangladesh A;Sylhet B;Barisal, C;Chittagong, D;Dhaka, E;Khulna, F;Rajshahi, and G;Rangpur, N.A.; Data not available

^a^Other category of Contraceptive use; condom, male sterilization, rhythm/periodic abstinence, withdrawal, lactational amenorrhea, female condom and foam or jelly

Table 2 exhibits the unadjusted percentage distribution of anaemia by different socioeconomic, demographic, health and food consumption covariates. Younger women (age group < 25 years), thin women (with BMI < 18), women with 2 + ever-born children (CEB), illiterate women, Hindu, poor, and rural women, women practices open defecation, and women with agricultural land suffered more from anaemia. This observation was evident in both the spatial units of comparison. In Bangladesh, households with food security were less likely to suffer from anaemia, whereas in West Bengal, better chicken or meat consumption reduced anaemia's prevalence significantly.

**Table 2:** Weighted Percentage distribution of anaemia among women by background characteristic in West Bengal and Bangladesh: NFHS 2015-16 and BDHS 2011

Background characteristics	West Bengal			Bangladesh		
	N	%	Chi	n	%	Chi
**Demographic and health factors**						
**Age**			12.81 ***			
Below 20	1,591	62.50		208	40.13	16.09 ***
20–24	1,654	61.74		383	39.88	
25–29	1521	59.79		355	38.35	
30&above	1,458	64.47		1,253	44.11	
**Body mass index**			131.44 ***			92.93 ***
Thin	2,317	68.63		694	51.29	
Normal	5,787	62.67		1,241	41.23	
Overweight or Obese	1,818	57.76		265	29.97	
**Current contraceptive method**			122.32 ***			26.16 ***
Not using	3,983	62.32		903	44.53	
Female sterilisation	2,763	68.49		124	47.21	
Pill/injection/IUD	1,554	56.63		773	37.78	
Others^a^	1,622	62.66		398	43.94	
**Children ever born**			39.74 ***			22.93 ***
Child	2,252	59.64		153	36.42	
1–2	5,111	63.06		905	38.84	
2 +	2,559	65.99		1,140	45.74	
**Socioeconomic factors**						
**Education**			88.04 ***			31.99 ***
Illiterate/primary	4,362	66.93		1,386	45.19	
Secondary	4,900	60.68		702	38.51	
Higher	661	56.66		111	31.33	
**Religion**			65.83 ***			19.45 ***
Hindu	7,582	65.19		264	50.42	
Muslim	2,340	56.68		1,935	40.98	
**Wealth**			141.56 ***			58.34 ***
Poor	5,451	66.46		965	48.42	
Middle	2,126	61.11		430	42.04	
Rich	2,345	57.52		804	36.07	
**Source of drinking water**						
Groundwater	7,025	65.62	35.48 ***	1,804	42.70	19.56 ***
Distributed water	2,796	60.15		180	30.98	
Others	212	51.70		195	41.49	
**Practicing open defecation**			54.15 ***			15.45 ***
Yes	2,700	69.91		95	53.05	
No	7,223	60.67		2,104	41.53	
**Has own land for agriculture**			62.90 ***			3.81 *
No	6,663	61.42		1,102	40.77	
Yes	3,259	66.38		1,097	43.15	
**Place of residence**			93.44 ***			21.72 ***
Urban	2,899	58.58		487	35.56	
Rural	7,024	64.97		1,712	44.18	
**Food related factors**						
**Food security**						
Secure	NA	NA		1,386	40.88	11.23 ***
Insecure	NA	NA		832	43.76	
**Consume pulses**			9.50 ***			
Daily	4,046	61.96		NA	NA	
Never/weekly/occasionally	5,877	63.97		NA	NA	
**Consume fruits**						
Daily	889	55.27		NA	NA	
Weekly	3,512	62.80	53.39 ***	NA	NA	
Never/occasionally	5,521	64.52				
**Consume chicken or meat**				NA	NA	
Daily/ weekly	5,009	61.92		NA	NA	
Occasionally	4,612	64.92	46.36 ***	NA	NA	
Never	302	67.65				

Significant level; *p < 0.10, **p < 0.05, ***p < 0.01

^**a**^Other category of Contraceptive use; condom, male sterilization, rhythm/periodic abstinence, withdrawal, lactation amenorrhea, female condom and foam or jelly

In Bangladesh, 46% of women, who had 2 + children ever born (CEB), were anaemic. A similar finding was observed among women of West Bengal, with the highest anaemia rate of 66% among women with 2 + CEB compared to those with no CEB (60%). The poor-rich gap in the prevalence of anaemia was equally prevalent in both places. In Bangladesh, the difference was about 12 percentage points, whereas, in West Bengal, it was 9 percentage points. The pattern of the gap in the anaemic population by education and by practicing versus not practicing open defecation group was similar in both countries.

### Determinants of anaemia: West Bengal vs Bangladesh

A logistic regression model investigates the determinants of anaemia among women in West Bengal and Bangladesh (Table 3). The adjusted odds ratio shows that adolescent women had a higher probability of having anaemia (OR, 1.23; 95% CI: 1.06–1.42) compared to women in the age group of 30 and above in West Bengal. However, such a finding was not observed for Bangladesh. In West Bengal as well as Bangladesh, thin (BMI < 18 kg/m^2^) women were more likely to be anaemic than women with a normal weight (West Bengal: OR, 1.22; 95% CI: 1.10–1.36; Bangladesh: OR, 1.39; 95% CI: 1.19–1.62). It was observed that contraceptive methods like pill, injection and IUD were significantly protective determinants of anaemia than the female sterilization in both the study areas. In West Bengal, the chances of having anaemia increased with the growing number of children a woman had.

**Table 3:** Odds Ratios explaining anaemia among women in West Bengal and Bangladesh: Result of logistic regression

Determinants	West Bengal	Bangladesh
**Demographic and health factors**		
**Age**		
30& above ®		
Below 20	1.23***(1.06 1.42)	1.01 (0.75 1.36)
20–24	1.13**(1.00 1.25)	0.99 (0.79 1.23)
25–29	1.01 (0.92 1.12)	0.90 (0.75 1.07)
**Body Mass Index**		
Normal ®		
Thin	1.22***(1.10 1.36)	1.39***(1.19 1.62)
Overweight/obese	0.87**(0.78 0.97)	0.64***(0.52 0.77)
**Current contraceptive method**		
Female sterilization ®		
Not Using	0.96 (0.86 1.08)	0.98 (0.72 1.33)
Pill/injection/IUD	0.68***(0.59 0.78)	0.71***(0.52 0.98)
Others^a^	0.95 (0.83 1.10)	0.98 (0.72 1.35)
**Children ever born**		
No child ®		
1–2	1.34***(1.15 1.54)	1.14 (0.86 1.51)
2 +	1.36***(1.13 1.63)	1.31* (0.94 1.82)
**Socioeconomic factors**		
**Education**		
Secondary ®		
Illiterate/primary	1.18***(1.04 1.38)	1.06 (0.90 1.22)
Higher	1.02 (0.91 1.21)	0.83 (0.62 1.10)
**Religion**		
Muslim ®		
Hindu	1.46***(1.31 1.61)	1.60*** (1.28 1.99)
**Wealth**		
Rich ®		
Poor	1.20***(1.05 1.38)	1.45***(1.18 1.77)
Middle	1.02 (0.95 1.16)	1.12 (0.92 1.38)
**Source of drinking water**		
Distributed water ®		
Groundwater	1.14***(1.05 1.23)	1.25**(1.02 1.52)
Others	0.74***(0.59 0.90)	1.29*(0.99 1.68)
**Practicing open defecation**		
No ®		
Yes	1.31 ***(1.201.42)	1.26 (0.92 1.71)
**Have agricultural land**		
No ®		
Yes	1.19***(1.08 1.31)	1.22***(1.05 1.40)
**Place of residence**		
Urban ®		
Rural	1.09 **(1.00 1.19)	1.00 (0.81 1.22)
**Food related factors**		
**Food security Index**		
Secure ®	NA	
Not secure	NA	1.12 **(0.99 1.21)
**Consume pulses**		
Daily ®		NA
Never/weekly/occasionally	1.02 (0.96 1.1)	NA
**Consume fruits**		NA
Daily ®		NA
Never/occasionally	1.16**(1.03 1.31)	NA
Weekly	1.21***(1.07 1.36)	NA
**Consume chicken or meat**		NA
Daily/ weekly ®		NA
Never	1.28**(1.06 1.56)	NA
Occasionally	1.06 (0.99 1.13)	NA
**Region**		
A ®		
B	1.10 (0.97 1.24)	1.50 ***(1.11 2.02)
C	1.06 (0.95 1.18)	1.20 (0.92 0.59)
D	1.09 (0.98 1.22)	1.53 ***(1.17 1.99)
E	1.17*** (1.04 1.31)	1.20 (0.89 1.63)
F		1.49 ***(1.12 1.99)
G		1.68 ***(1.27 2.20)

Reference category; ® and significant level; *p < 0.10, **p < 0.05, ***p < 0.01

Administrative regions of west Bengal in the table A; Jalpaiguri, B;Burdwan, C;Presidency, D;Maldah, E;Medinipur and in Bangladesh A;Sylhet B;Barisal, C;Chittagong, D;Dhaka, E;Khulna, F;Rajshahi, and  G;Rangpur

^a^Other category of Contraceptive use; condom, male sterilization, rhythm/periodic abstinence, withdrawal, lactational amenorrhea, female condom and foam or jelly. N.A.; Data not available

Compared to higher educated women, illiterate or primary educated women were significantly more associated with anaemia in West Bengal (OR, 1.18; 95% CI, 1.04–1.38). Amongst Hindus, the odds of anaemia were greater (OR, 1.46; 95% CI, 1.31–1.61) compared to Muslims in West Bengal, with the magnitude being much higher in Bangladesh (OR, 1.60; 95% CI, 1.28–1.99). Women who belonged to the poor wealth quintile had a larger probability of being anaemic than those who were rich (West Bengal: OR, 1.20; 95% CI: 1.05–1.38; Bangladesh: OR, 1.45; 95% CI: 1.18–1.77). In West Bengal, women who lived in rural areas (OR, 1.09; 95% CI: 1.00–1.19) and practiced open defecation (OR, 1.31; 95% CI: 1.20–1.42) were more likely to be anaemic than their counterparts, i.e., women who lived in urban areas and practiced better defecation systems. Women who had their own agricultural land suffered more from anaemia compared to those who did not own any agricultural land in both places under the study. Using groundwater (tube well or well) for drinking was a major risk factor for anaemia in both Bengals (West Bengal: OR, 1.14; 95% CI: 1.05–1.23; Bangladesh: OR, 1.25; 95% CI: 1.02–1.52) compared to women who used distributed water (tank, tap, and piped water) as the source of drinking water.

**Fig. 3:**
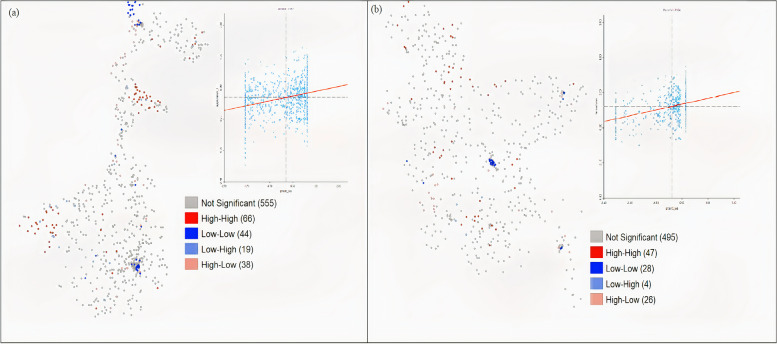
Bivariate LISA map showing the spatial correlation between drinking groundwater and anaemia among women in Wes Bengal **(a)** and Bangladesh (**b**)

The paper emphasized the spatial correlation between drinking groundwater and anaemia. The bivariate LISA map of our study shows a significant spatial correlation between drinking groundwater and anaemia (Fig. 3) in both Bengals (West Bengal: β = 0.15; Bangladesh: β = 0.16).

In Bangladesh, women who lacked food security had a higher odds of anaemia (OR, 1.12; 95% CI: 0.99–1.21). In West Bengal, women who never or weekly consumed fruits (OR, 1.21; 95% CI: 1.07–1.36) and those who never consumed chicken or meat (OR, 1.28; 95% CI: 1.06–1.56) were more likely to be anaemic. The women residing in Barisal, Rajshahi, Rangpur, and Dhaka had significantly higher chances of being anaemic in Bangladesh; in West Bengal, women in the Medinipur region had a higher likelihood of having anaemia.

Table 4 shows religious effects on the chances of anaemia. The highest odds of anaemia were observed among Hindu women in both Bengals (West Bengal: OR, 2.85; 95% CI: 2.55–3.18; Bangladesh: OR, 1.58; 95% CI: 1.26–1.96). However, within the 'Hindu' group, the probability of being anaemic was higher in West Bengal. Even among Muslims, anaemia was more prevalent in West Bengal than in Bangladesh (OR, 1.93; 95% CI: 1.70–2.19).

**Table 4:** Odds Ratios explaining effects of religion on anaemia among women in India and Bangladesh: Result of logistic regression

Determinants	OR
**Demographic and health factors**	
**Age**	
30& above ®	
Below 20	1.20 *(1.02 1.41)
20–24	1.09 (0.95 1.15)
25–29	0.93 (0.86 1.02)
**Body Mass Index**	
Normal ®	
Thin	1.25***(1.13 1.37)
Overweight/obese	0.84***(0.76 0.94)
**Current contraceptive method**	
Female sterilization ®	
Not Using	0.97 (0.82 1.02)
Pill/ injection/IUD	0.69***(0.61 0.78)
Others^a^	0.96 (0.80 1.04)
**Children ever born**	
No child ®	
1–2	1.30***(1.14 1.48)
2 +	1.35**(1.14 1.59)
**Socioeconomic factors**	
**Education**	
Secondary ®	
Illiterate/primary	1.17***(1.06 1.28)
Higher	1.01 (0.9 1.14)
**Wealth**	
Rich ®	
Poor	1.24***(1.10 1.41)
Middle	1.05(0.93 1.18)
**Source of drinking water**	
Distributed water ®	
Groundwater	1.08***(1.02 1.13)
Others	0.65 ***(0.56 0.75)
**Practicing open defecation**	
No ®	
Yes	1.34 ***(0.1.23 1.44)
**Have agricultural land**	
No ®	
Yes	1.19***(1.09 1.29)
**Place of residence**	
Urban ®	
Rural	1.04 (0.94 1.16)
**Interaction of Country &Religion**	
Bangladesh × Muslim ®	
Bangladesh × Hindu	1.58***(1.26 1.96)
West Bengal × Hindu	2.85***(2.55 3.18)
West Bengal × Muslim	1.93***(1.70 2.19)

Reference category; ® and significant level; *p < 0.10. **p < 0.05. ***p < 0.01

^a^Other category of Contraceptive use; condom, male sterilization, rhythm/periodic abstinence, withdrawal, lactational amenorrhea, female condom and foam or jelly

## Discussion

The comparative study on anaemia outcomes among women was carried out to identify the potential determinants in two resource-limited areas, i.e., West Bengal and Bangladesh, where similar ethnic and environmental parameters can be found. This natural control of the population may help us identify some unique features explaining women's anaemia. The results may help in developing strategies and attaining SDG 2.

Using the large-scale Demographic Health Surveys (DHS) of both the areas under study, the research reveals some pertinent results that needs policy attention.

First, higher prevalence of anaemia in West Bengal, India against Bangladesh is well observed. The hotspots of the prevalence of anaemia were concentrated in the Dakshin Dinajpur district, West and North-Eastern West Bengal. In Bangladesh, people who are living in the surrounding areas of Jamuna and Padma rivers (boundary of Rangpur and Dhaka, and Barisal) are highly exposed to arsenic-contaminated groundwater, that is well observed in our study [41–43].

Second, the concentration of anaemia in these above mentioned areas can be corroborated well with ground water use and poverty. Using groundwater (tube well or well) for drinking was found to be a significant risk factor for anaemia in this study. Previous researches have indicated that fluorosis contamination in groundwater is heavily concentrated, as is poverty in the western part of West Bengal [44–46]. Most people (90%) of the Dakshin Dinajpur district drink groundwater where arsenic is highly concentrated [4]. Further, the tribal population living in the West and North-Eastern parts of West Bengal are more vulnerable to anaemia due to thalassemia and nutrient deficiency [47–49]. West Bengal and neighbouring Bangladesh have the highest reported population of exposure to inorganic arsenic. In 2015, more than 50 million people in West Bengal and 77 million people in Bangladesh were exposed to more than 0.01 mg of arsenic in drinking water [43]. Among 23 districts of West Bengal, nine districts encompassing about 38,861 sq. km were identified as highly affected by arsenic [50]. Previous studies have demonstrated that exposure to arsenic and fluoride-contaminated drinking water increases the risk of anaemia [42, 45, 51]. In countries like India and Bangladesh, most people drink water directly collected from tube wells or wells without purification, increasing the risk of drinking contaminated water [52]. In line with the hypothesis, the bivariate LISA map of our study shows a significant spatial correlation between using groundwater and anaemia. The spatial clustering can be observed in the areas with highly contaminated groundwater identified by the previous studies [43, 53–56]. Thus, the study concludes that most people who collect drinking water from tube wells or wells in highly groundwater-contaminated areas experience more anaemia. However, an in-depth study is necessary to strengthen this finding.

Third, open defecation is taking a significant role in risk of anaemia in West. Bangladesh has meaningfully addressed open defecation in the recent past. A significant proportion of the population in West Bengal practice open defecation, especially among Hindus than Muslims. Practicing open defecation is a leading factor of parasitic infection which damages the intestinal walls, causes blood loss and reduces the absorption capacity of nutrients from food [57, 58]. Government of India launched the 'Swachh Bharat Mission' in 2014, intending to make India open defecation-free by building new toilets in every household [59]. Moreover, six SDG envisages achieving access to adequate and equitable sanitation and hygiene for all and ending open defecation [60]. Nevertheless, people prefer open defecation even after having a latrine, as reported by the SQUAT (Sanitation Quality, Use, Access, and Trends) survey [61]. Thus, increasing awareness regarding the adverse impact of open defecation is necessary to bring a change in the behaviour of people.

Fourth, being undernourished and belonging to the low wealth quintile are major contributors to anaemia in both the places. At the same time, the household wealth quintile is an independent factor of undernourishment due to inadequate food consumption [62]. Malnutrition may not be directly linked to anaemia, however, it increases the likelihood having a weaker immune system that leads various health problems such as parasitic infections or chronic inflammation [63]. These are responsible for reducing the level of haemoglobin in the blood [64].

Fifth, in West Bengal, those who regularly consume fruits, chicken or meat are less likely to be anaemic. A poor woman being unable to afford micronutrient-rich food makes her more vulnerable to anaemia than a woman from a wealthy family. There is also evidence that poor households are unable to utilize health care facilities due to the lack of accessibility and affordability [65, 66]. Thus, there is a possibility of diseases remaining untreated that can cause anaemia.

Sixth, households with agricultural land indicate more anaemia in our study. The explanations for such finding are complex and need further investigation. Needless to mention, women's participation is rapidly increasing in the agricultural sector. Women in India represent 33% of the working population in agriculture and 48% of independent farmers [67]. In Bangladesh, 50% of women are engaged in the agricultural sector [68]. In lower- and middle-income countries women's participation in agriculture negatively impacted their nutritional status due to the time trade-offs with food preparation in the household [69], which supports the findings of the present study.

Seventh, less education and a higher number of children trigger the risk of anaemia in West Bengal. In Bangladesh, education and rural stay have no distinct differential effect on anaemia, indicating a similar distribution of non-anaemic women across age, education and place of residence. Initially, the pregnant and lactating women and preschool children between 1 and 5 years were targeted by the anaemia schemes in India. Now, such schemes are expanded and all children above six months to adults are being covered. Due to the large targeted population, a huge iniquity and problems related to the scheme's coverage exists [70]. Thus, better monitoring is needed to improve the performance of the nutritional anaemia programme in both countries. This is true that women with higher education have higher chances of utilizing health care facilities than non-educated women and obtaining preventive and remedial services for diseases that improve anaemia levels [65, 71]. The probability of receiving IFA tablets too increases with increasing educational levels as educated women are aware of the importance of iron to the human body [72, 73]. In the present study, giving three or more births was found to be a great risk for anaemia, as supported by previous studies [26, 74].

Eighth, adolescents have a greater probability of being anaemic as compared to the older women (aged 30 & above) in West Bengal, while in Bangladesh, we observed no such age effect. Calorie, protein and mineral requirements increase significantly during adolescence due to the rapid somatic growth and increase the red blood cell mass [75]. Moreover, adolescents in South East Asian countries don't consume sufficient iron-rich foods [76]. Higher demand for iron is imposed on a woman due to the iron loss during menstruation [77]. A previous study from India noted that about 30% of adolescent girls reported abundant blood loss during their menstrual period, making it a major contributor to anaemia [78]. Dietary modification through spreading awareness or nutritional schemes like mid-day meals along with preventive supplementation is required to improve the haemoglobin status of the adolescents.

Ninth, using IUD, pill, or injection reduces chances of anaemia, which is in line with previous studies [79, 80]. Does it mean that sterilization prevalent in West Bengal is taking a toll on women in terms of anaemia? Studies are limited in this direction. Using hormonal methods gives women better haemoglobin concentration as compared to sterilization [80]. A past study proved that hormonal and IUD methods protect women from menstrual blood loss [81]. In contrast, female sterilization carries the risk of women developing polymenorrhagia, hypermenorrhoea, menorrhagia and an irregular menstrual cycle that triggers anaemia. In West Bengal, around 29% of women had undergone female sterilization in 2015–16 [4], whereas only 5% of women were sterilized in Bangladesh in 2017–18 [82]. Therefore, supporting couples and individuals to decide freely and responsibly on contraceptive use (method choice) could explain the lower prevalence of anaemia in Bangladesh. The current approach of family planning programs in India focuses on addressing unmet needs, while female sterilization remains in high demand [83]. In this context, Bangladesh has drastically declined female sterilization by promoting basked of choices in modern contraceptive methods [84]. Thus, a wider choice of contraception is essential in West Bengal.

Tenth, in West Bengal, it is observed that consuming meat or chicken and fruits were prospective habits of anaemia. Previous studies observed that high fruit consumption not only improves micronutrient concentration but also significantly protects from chronic diseases [19, 85]. Therefore, those who consume more fruit are more likely to have increased iron absorption, which reduces their risk of anaemia [86]. Here, religion plays a significant role in the selection of foods. We found that Hindu women were more vulnerable to anaemia in both Bengals. About 30% of women are vegetarian in India [4]. A study in India found that the incidence of undernutrition and iron deficiency anaemia is higher among Hindu women than their Muslim counterparts [87]. Also, the vegetarian diet was found to be a factor responsible for developing an iron deficiency. Consuming meat helps to improve haemoglobin [88]. In contrast to India, 90% of the population of Bangladesh belongs to the Muslim religion, and they consume non-vegetarian foods. People of Bangladesh consume 11.96 g/day/person of animal protein [89], which is almost double than that in India (6.16 g/day/person) [89, 90]. There is a vitamin B12 deficiency in 51% of pregnant Hindu women in India [91]. However, in comparison with Muslims, higher proportions of Hindus in both places practice open defecation and female sterilization methods (Supplementary).

Interestingly, Hindus are more anaemic in both the study regions, and women of West Bengal are more anaemic, irrespective of religion, compared to the women of Bangladesh in the interaction model. It proves that area-specific intervention could address anaemia by considering the religious backdrop. Encouraging a diversified protein and micronutrient diet might be a great strategy to supply the essential nutrients, especially for vegetarians. These shreds of evidence might partly explain the prevalence gap of anaemia among women in West Bengal and Bangladesh.

### Limitations

The study has some limitations.  Wealth categories are relative; NFHS and BDHS calculated the wealth quintiles as per the amenities of a household in the given country. Also, the nutritional deficiencies related to anaemia may coexist with abnormal haemoglobin variants, complicating their diagnosis. DHS does not capture clinical, haematological, and biochemical diagnoses of anaemia. Further, the study compared the 6^th^ round of BDHS, 2011 and 4th round of NFHS, 2015-16, as the information of haemoglobin was not available in the recent dataset of BDHS 2017-18. The study also overlooked the possibility that a woman may remain a nonuser of contraception because she suffers from anaemia. Similarly, lower BMI could be due to anaemic conditions.

## Conclusions

By identifying some unique explanatory factors, the study makes an important contribution to the literature on anaemia among women. To our knowledge, this is the first empirical study that compares two naturally controlled spaces to find out the potential risk factors of anaemia among women in West Bengal and Bangladesh using nationally representative surveys.

Use of a particular method of contraception, food consumption pattern/food security, water and sanitation well explain the difference in the prevalence of anaemia in West Bengal and Bangladesh, besides the usual determinants like wealth, education and fertility. Bangladesh, due to its unique advantages in some of the factors mentioned above, like better sanitation and hormonal contraception, is more efficient in tackling women's anaemia than West Bengal, though the former experience higher regional variation in anaemia. Promoting knowledge and access to nutrient-rich foods grown in the local environment is necessary to tackle anaemia. Further, awareness of anaemia and associated health problems can encourage women to choose suitable methods of contraception, small family norm, safe sanitation and safe drinking water use. The national focus is essential toward healthy food consumption, better economic condition and higher education for women. Policies must target the provision of safe drinking water and open defecation-free environment, especially in West Bengal and to create a secure food community mainly in Bangladesh to reduce anaemia.

## Supplementary Information

**Additional file 1:** **Table S1.** Background characteristics of study population by Hindu and Muslim.

## Data Availability

The study uses secondary data that are available on reasonable request through https://dhsprogram.com/data/dataset_admin/
