# Supplementary material for: Identifying risk factors in explaining women’s anaemia in limited resource areas: evidence from West Bengal of India and Bangladesh
Source: BMC Public Health. 2022 Jul 28;22:1433. doi: 10.1186/s12889-022-13806-5 (PMC9330636; doi:10.1186/s12889-022-13806-5)
Supplement: Supplementary file 1 — Additional file 1: Table S1. Background characteristics of study population by Hindu and Muslim. [file 12889_2022_13806_MOESM1_ESM.docx]

Table S1. Background characteristics of study population by Hindu and Muslim.

| **Background Characterises** | **West Bengal** | |  | **Bangladesh** | |
| --- | --- | --- | --- | --- | --- |
|  | % Hindu | %Muslim |  | % Hindu | %Muslim |
| **Age** |  |  |  |  |  |
| Below 20 | 15.23 | 20.65 |  | 7.58 | 10.45 |
| 20-24 | 15.98 | 19.34 |  | 15.99 | 18.01 |
| 25-29 | 15.52 | 15.66 |  | 17.51 | 18.27 |
| 30&above | 53.27 | 44.35 |  | 58.92 | 53.27 |
| **Body mass index** |  |  |  |  |  |
| Thin | 23.26 | 23.19 |  | 25.08 | 24.90 |
| Normal | 58.92 | 59.09 |  | 56.06 | 57.15 |
| Overweight or Obese | 17.82 | 17.78 |  | 18.86 | 17.95 |
| **Current contraceptive method** |  |  |  |  | 34.34 |
| Not using | 39.23 | 50.31 |  |  | 38.93 |
| Female sterilization | 29.92 | 14.35 |  | 6.40 | 4.63 |
| Pill/injection\IUD | 14.64 | 20.47 |  | 36.53 | 26.09 |
| Others * | 16.22 | 14.86 |  | 22.73 | 30.36 |
| **Children ever born** |  |  |  |  |  |
| No child | 22.93 | 27.55 |  | 6.90 | 8.57 |
| 1-2 | 54.40 | 37.14 |  | 54.55 | 44.25 |
| 2+ | 22.67 | 35.31 |  | 38.55 | 47.18 |
| **Socioeconomic factors** |  |  |  |  |  |
| **Education** |  |  |  |  |  |
| Illiterate/primary | 42.28 | 48.45 |  | 52.69 | 57.62 |
| Secondary | 50.20 | 47.52 |  | 38.72 | 35.02 |
| Higher | 7.52 | 4.03 |  | 8.59 | 7.35 |
| **Wealth** |  |  |  |  |  |
| Poor | 52.43 | 62.40 |  | 36.03 | 35.83 |
| Middle | 21.94 | 22.06 |  | 20.03 | 19.10 |
| Rich | 25.62 | 15.54 |  | 43.94 | 45.07 |
| **Source of drinking water** |  |  |  |  | 7.91 |
| Distributed water | 30.83 | 25.48 |  |  | 11.40 |
| Groundwater | 66.01 | 73.56 |  | 83.67 | 79.60 |
| Others | 3.16 | 0.96 |  | 8.42 | 9.00 |
| **Practicing open defecation** |  |  |  |  | 9.60 |
| Yes | 30.29 | 26.49 |  |  | 2.56 |
| No | 69.71 | 73.51 |  | 90.40 | 97.44 |
| **Has own land for agriculture** |  |  |  |  | 42.93 |
| Yes | 34.83 | 32.56 |  |  | 48.03 |
| No | 65.17 | 67.44 |  | 57.07 | 51.97 |
| **Place of residence** |  |  |  |  |  |
| Urban | 28.07 | 24.88 |  | 30.81 | 35.51 |
| Rural | 71.93 | 75.12 |  | 69.19 | 64.49 |
| **Food related factors** |  |  |  |  |  |
| **Food security** |  |  |  |  |  |
| Secure |  |  |  | 66.50 | 64.44 |
| Insecure |  |  |  | 33.50 | 35.56 |
| **Consume pulses** |  |  |  |  |  |
| Daily | 44.89 | 38.08 |  |  |  |
| Never/weekly/occasionally | 55.11 | 61.92 |  |  |  |
| **Consume fruits** |  |  |  |  |  |
| Daily | 9.19 | 8.69 |  |  |  |
| Never/occasionally | 55.30 | 56.74 |  |  |  |
| Weekly | 35.50 | 34.58 |  |  |  |
| **Consume chicken or meat** |  |  |  |  |  |
| Daily/ weekly | 48.69 | 59.98 |  |  |  |
| Never | 3.89 | 1.51 |  |  |  |
| Occasionally | 47.42 | 38.50 |  |  |  |
